# Long non-coding RNA Taurine upregulated gene 1 promotes osteosarcoma cell metastasis by mediating HIF-1α via miR-143-5p

**DOI:** 10.1038/s41419-019-1509-1

**Published:** 2019-03-25

**Authors:** Xiao Yu, Lei Hu, Suoyuan Li, Jun Shen, Donglai Wang, Renjie Xu, Huilin Yang

**Affiliations:** 1 Department of Orthopedics, The first affiliated hospital of Soochow University, Suzhou, People’s Republic of China; 2 Department of Orthopedics, Suzhou Municipal Hospital, The Affiliated Suzhou Hospital of Nanjing Medical University, Suzhou, People’s Republic of China; 3 Department of General Surgery, Anhui Provincial Hospital, Anhui Medical University, Hefei, People’s Republic of China

## Abstract

Early aggressive metastasis of osteosarcoma (OS) leads to rapid progression and poor prognosis. Increasing evidence has demonstrated that long non-coding RNAs (lncRNAs) could serve as crucial regulators to modulate tumour metastasis. In this study, we reported the critical role of lncRNA TUG1 in determining OS metastasis. TUG1 was significantly upregulated in OS tissues and associated with tumour size, distant metastasis, TNM stage, and overall and recurrence-free survival, which further indicated poor prognosis. Furthermore, CAFs-derived TGF-β could upregulate TUG1 expression, and the crosstalk between CAFs and OS cells induced TUG1 to promote OS cell metastasis. Dysregulated TUG1 expression could act as an miRNA “sponge” to competitively protect the HIF-1α mRNA 3′UTR from miR-143-5p. Our study emphasised the effects of TUG1 in OS and demonstrated a novel axis by which TUG1 regulated OS cell metastasis, angiogenesis, and proliferation in vivo and in vitro. Collectively, TUG1 might be a prognostic indicator for OS and could be a therapeutic target for OS.

## Introduction

Among deadly tumours, osteosarcoma (OS) remains a major threat due to its malignant phenotype in children and healthy young people. Early aggressive metastasis of OS leads to rapid progression and poor prognosis. Due to frequent pulmonary metastasis, the 5-year survival rate of OS patients with metastasis is lower than 35%^1,2^. Obviously, understanding the mechanism and blocking tumour metastasis are ideal strategies and preferred applications to improve OS patients’ survival rates and prognosis. Several studies have investigated the underlying mechanism involved in OS progression and recurrence^3^; however, the crucial molecular mechanism behind OS metastasis remains largely obscure. Therefore, it is urgent to explore potential molecular mechanisms of OS progression and metastasis, which could improve prognosis of OS patients.

Tumour metastasis pathway, including epithelial-mesenchymal transition (EMT), invasion, migration, and angiogenesis, is involved in multiple and complex crosstalk networks of diverse genes^4–6^. In addition, cytokines derived from cancer-associated fibroblasts (CAFs) in the tumour microenvironment have significant effects on gene expression and tumour metastasis^7^. Increasing evidence has demonstrated that long non-coding RNAs (lncRNAs) could serve as crucial regulators to modulate the tumour metastasis-associated pathway at the epigenetic, transcription, or post-transcription levels. MEG3 was found to be inversely associated with VEGF levels, which is involved in angiogenesis in osteoarthritis^8^. Silencing of HULC inhibited angiogenesis by suppressing invasion via the AKT/mTOR pathway, which was positively associated with VEGF and micro-vessel density in gliomas^9^. Taurine upregulated gene 1 (TUG1) has been shown to act as a potential oncogene and drew our attention, in which it was reported to have dysregulated expression in OS and association with distant metastasis, indicating poor survival rates^10^. With the widespread acceptance of the competitive endogenous RNA (ceRNA) hypothesis, reciprocal repression between lncRNAs and miRNAs was investigated to uncover the potential mechanism of metastasis in malignant tumours. However, underlying molecular mechanisms of TUG1 in OS metastasis remain unknown.

In the present study, our results suggested that TUG1 was significantly upregulated in OS tissues, which also indicated poor prognosis in patients with OS. Furthermore, CAFs-derived TGF-β could upregulate TUG1 expression, and the crosstalk between CAFs and OS cells induced TUG1 to promote OS cell metastasis. Dysregulated TUG1 expression could act as a miRNA “sponge” to competitively protect HIF-1α mRNA 3′UTR from miR-143-5p, and elevated TUG1 could promote OS cell migration, invasion, and angiogenesis in vivo and in vitro.

## Materials and methods

### Tissue samples

Human OS tissues and the corresponding para-tumour tissues used in this study were obtained from the Department of Orthopedics, Suzhou Municipal Hospital, The Affiliated Suzhou Hospital of Nanjing Medical University from March 2009 to February 2012. Written informed consent was obtained from all participants. No patient had received preoperative chemotherapy and radiotherapy. Each OS case was confirmed by a definite pathological diagnosis and staged by the TNM classification. Additionally, this study was approved by the Ethics Committee of Suzhou Municipal Hospital, The Affiliated Suzhou Hospital of Nanjing Medical University.

### Cell lines and culture

Human OS cell lines (143B, HOS, MG-63, Saos-2, and U2OS) and the normal human osteoplastic cell line NHOst were purchased from the American Type Culture Collection (ATCC, USA). 143B cells were cultured in DMEM/F12 medium (Gibco, USA), HOS and MG-63 cells in MEM medium (Gibco, USA), Saos-2 and U2OS cells in α-MEM medium (Gibco, USA), and NHOst cells in DMEM medium (Gibco, USA) at 37 °C in 95% air and 5% CO_2_. The recombinant human transforming growth factor-β (hTGF-β) used in this study was purchased from PeproTech, USA. CAFs were isolated from freshly resected human OS tissues at the Department of Orthopedics, Suzhou Municipal Hospital, The Affiliated Suzhou Hospital of Nanjing Medical University. Tumour tissues and adjacent non-tumour tissues (separated from the margin of the tumour resection by at least 5 cm) were mechanically minced into small pieces (1–1.5 mm^3^) and seeded onto 10 cm petri dishes in RPMI 1640 medium (Gibco, USA) containing 10% FBS (Gibco, USA). After 7–14 days of culture, these conditions would produce a homogeneous group of fibroblasts in the dishes. In order to minimize clonal selection and culture stress, we passaged the fibroblasts over 10 times and then used them for subsequent experiments. In addition, we performed an identification test of the harvested fibroblasts by western blot and immunofluorescence analysis using anti-human α-SMA (Cell Signaling Technology, USA) and FAP (Abcam, USA) antibodies. After co-culturing for 48 h, OS cells were harvested for subsequent experiments. The verification of CAFs and NFs is shown in Supplementary Figure 1.

### Quantitative real-time PCR (qRT-PCR)

Total RNA was extracted from OS tissues, corresponding para-tumour tissues, and OS cells using the Trizol reagent (Invitrogen, USA) according to the manufacturer’s protocol. RNA was reverse-transcribed into cDNAs using the Hifair™ III 1st Strand cDNA Synthesis Kit (Yeasen, China) or miRNA First Strand cDNA Synthesis Kit (Sangon Biotech, China). The mRNA or lncRNA level was assessed the using Hieff™ qPCR SYBR^®^ Green Master Mix Kit (Yeasen, China), and the miRNA level was assessed using the miRNAs qPCR Kit (Sangon Biotech, China) and ABI7500 system (Applied Biosystems, USA). GAPDH or small nuclear RNA U6 served as the internal control. The relative expression levels were calculated using the 2^-ΔΔCt^ method. The primers sequences are listed in Supplementary Table 1.

### Enzyme-linked immunosorbent assay (ELISA)

The levels of TGF-β in serum and tissue lysates of OS patients and OS cell lysates were assessed using an ELISA kit (R&D Systems, USA) according to the manufacturer’s protocol.

### RNA interference

Small interfering RNAs (siRNAs) that specifically target human TGF-β, TUG1, and HIF-1α were purchased from GenePharma, China. MiR-143-5p mimic, miR-143-5p inhibitor, and miR-NC were purchased from GenePharma, China. The pcDNA-TUG1 and empty vectors were purchased from Sangon Biotech, China. Transfection was conducted using Lipofectamine 2000 (Invitrogen, USA) according to the manufacturer’s protocol. The RNA interference sequences are listed in Supplementary Table 1.

### Western blot

Total protein of OS cells was extracted using RIPA lysis buffer (Beyotime, China) containing 1% PMSF (Beyotime, China). The primary antibodies used were human antibody TGF-β, HIF-1α, E-cadherin, N-cadherin, Vimentin, Slug, Twist, and GAPDH (Cell Signaling Technology, USA).

### Dual-luciferase reporter assay

In brief, cells were co-transfected with miR-143-5p mimic or miR-NC and empty pmiR-GLo, pmiR-Glo-TUG1-mut, or pmiR-Glo-TUG1-wt. After transfected for 48 h, cells were collected and lysed for luciferase assessment. The relative luciferase activity was assessed using a luciferase assay kit (Promega, USA) according to the manufacturer’s protocol and normalised to the *Renilla* luciferase activity.

### RNA immunoprecipitation (RIP) assay

RIP assay was performed using the EZ-Magna RIP RNA-Binding Protein Immunoprecipitation Kit (Millipore, USA) according to the manufacturer’s protocol. Briefly, cells were incubated with the RIP buffer containing magnetic beads coated with human Ago2 antibody (Cell Signaling Technology, USA). Normal mouse IgG (Cell Signaling Technology, USA) acted as a negative control, and SNRNP70 (Millipore, USA) acted as a positive control. The coprecipitated RNAs were isolated and assessed by qRT-PCR.

### Pull-down assay with Biotin-labelled miRNA

In brief, cells were transfected with biotinylated miR-143-5p, biotinylated miR-143-5p-mut, and biotinylated NC (GenePharma, China). After 48 h, the cell lysates were incubated with M-280 streptavidin magnetic beads (Invitrogen, USA) and processed as previously described^11^. The bound RNAs were isolated and assessed by qRT-PCR.

### Transwell invasion assay

The invasive ability of OS cells was assessed using 8-μm transwell chambers pre-coated with Matrigel (BD Biosciences, USA) and performed as previously described^12^.

### Tube formation assay

The tube formation assay was performed as previously described^13^.

### Cell counting kit-8 (CCK-8) assay

Cell viability was assessed using the CCK-8 (Dojindo, Japan) kit according to the manufacturer’s protocol. In brief, cells (1000 cells per well) were seeded into 96-well plates, CCK-8 solution was added every 24 h and incubated. Following this, the absorbance was measured at 450 nm.

### In vivo metastasis and tumourigenesis

All experiments on animal of this study were approved by the Committee on Animal Care in Nanjing Medical University. OS cells after transfection were injected subcutaneously, intraperitoneally, and intravenously via the tail vein into four-week-old male nude mice. Tumour sizes were assessed (0.5 × length × width^2^) every 3 days. Tumour metastasis nodules were calculated and analysed after sacrifice.

### Statistical analysis

All experiments were performed at least three times independently, and the data are presented as mean ± SD. Differences between groups were analysed using paired-samples *t*-test or independent-samples *t* test. The survival between groups was analysed using the Kaplan–Meier method and log-rank test. All statistical analyses were performed using GraphPad Prism 5.0. *P* *<* 0.05 was deemed statistically significant.

## Results

### Overexpression of TUG1 is associated with poor prognosis in OS patients

**Fig. 1:**
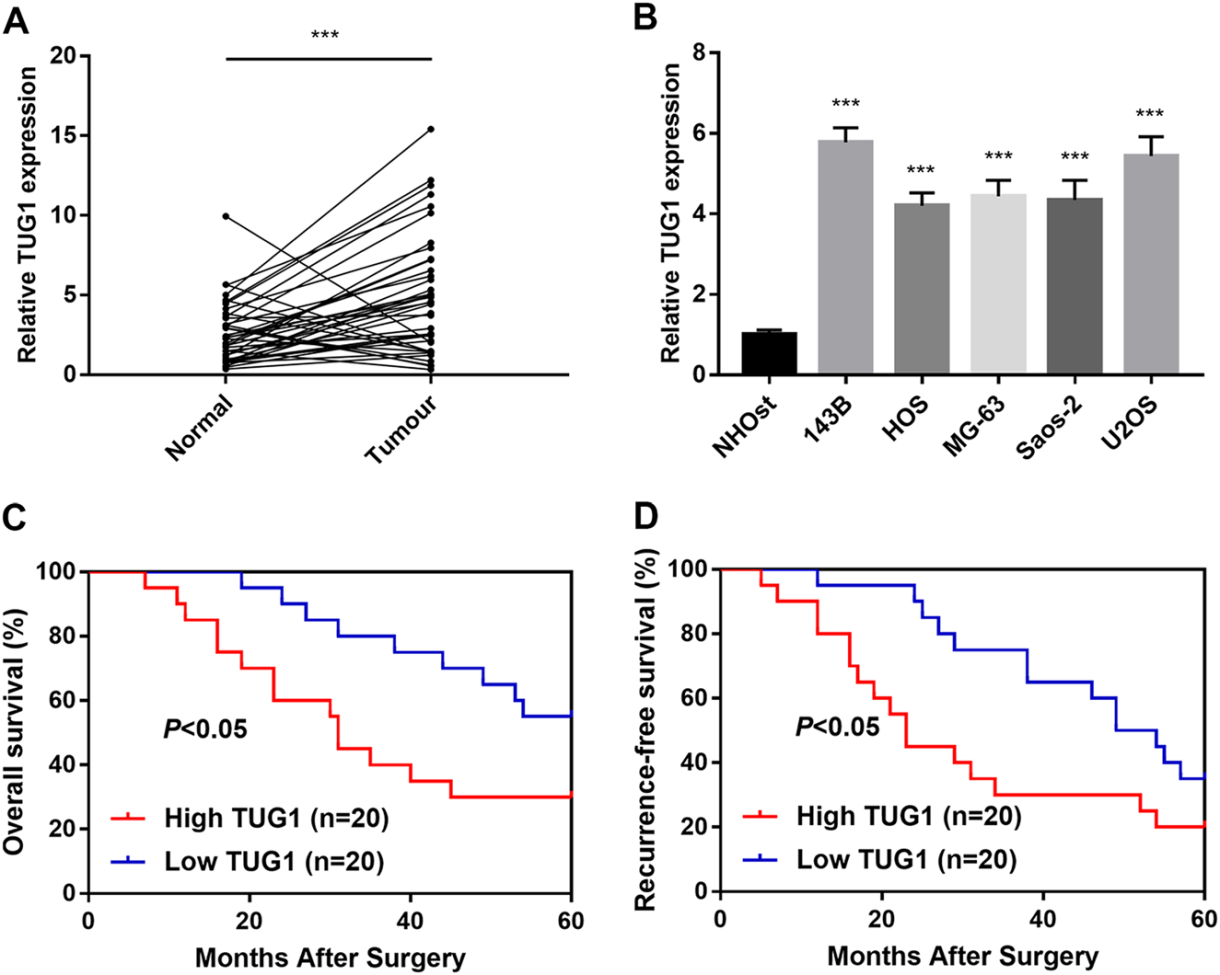
Overexpression of TUG1 is associated with poor prognosis in OS patients. **a** TUG1 expression in 40 pairs of human OS tissues and adjacent non-tumour tissues was quantified by qRT-PCR. **b** TUG1 expression levels in OS cell lines were quantified by qRT-PCR. **c** Overall survival of OS patients with relatively high and low expression of TUG1. **d** Recurrence-free survival of OS patients with relatively high and low expression of TUG1. ***P *<* 0.001

To better clarify the role of TUG1 in OS, quantitative real-time PCR was performed to evaluate TUG1 expression in OS tissues compared with normal ones. We found that TUG1 expression was significantly higher in OS tissues (Fig. 1a, *P* *<* 0.05). Furthermore, TUG1 was significantly upregulated in 143B, Saos-2, MG-63, U2OS, and HOS cell lines (Fig. 1b, *P* *<* 0.05). In addition, Kaplan–Meier survival analysis indicated that the overall survival rates and recurrence-free survival rate of patients with high TUG1 expression were significantly lower than those with low expression (Fig. 1c, d, *P* *<* 0.05). Statistical analysis suggested that TUG1 expression was significantly related to tumour size, distant metastasis, and TNM stage (Table 1). Thus, expression of TUG1 was significantly elevated, which is associated with poor prognosis in OS patients.

**Table 1:** The association of TUG1 expression in forty osteosarcoma patients with clinicopathologic characteristics

Characteristics	Case number	TUG1 expression		*P* value
		High (*n* = 20)	Low (*n* = 20)	
*Gender*				0.204
Male	18	7	11	
Female	22	13	9	
*Age at diagnosis*				0.516
≤18	23	12	11	
>18	17	8	9	
*Tumour size*				0.011*
≤8 cm	18	5	13	
>8 cm	22	15	7	
*Distant metastasis*				0.013*
No	29	11	18	
Yes	11	9	2	
*Anatomic location*				0.376
Tibia/femur	34	18	16	
Elsewhere	6	2	4	
*TNM stage*				0.027*
I + IIA	19	6	13	
II B/III	21	14	7	

*P < 0.05

### CAFs-derived TGF-β promotes TUG1 expression in OS

**Fig. 2:**
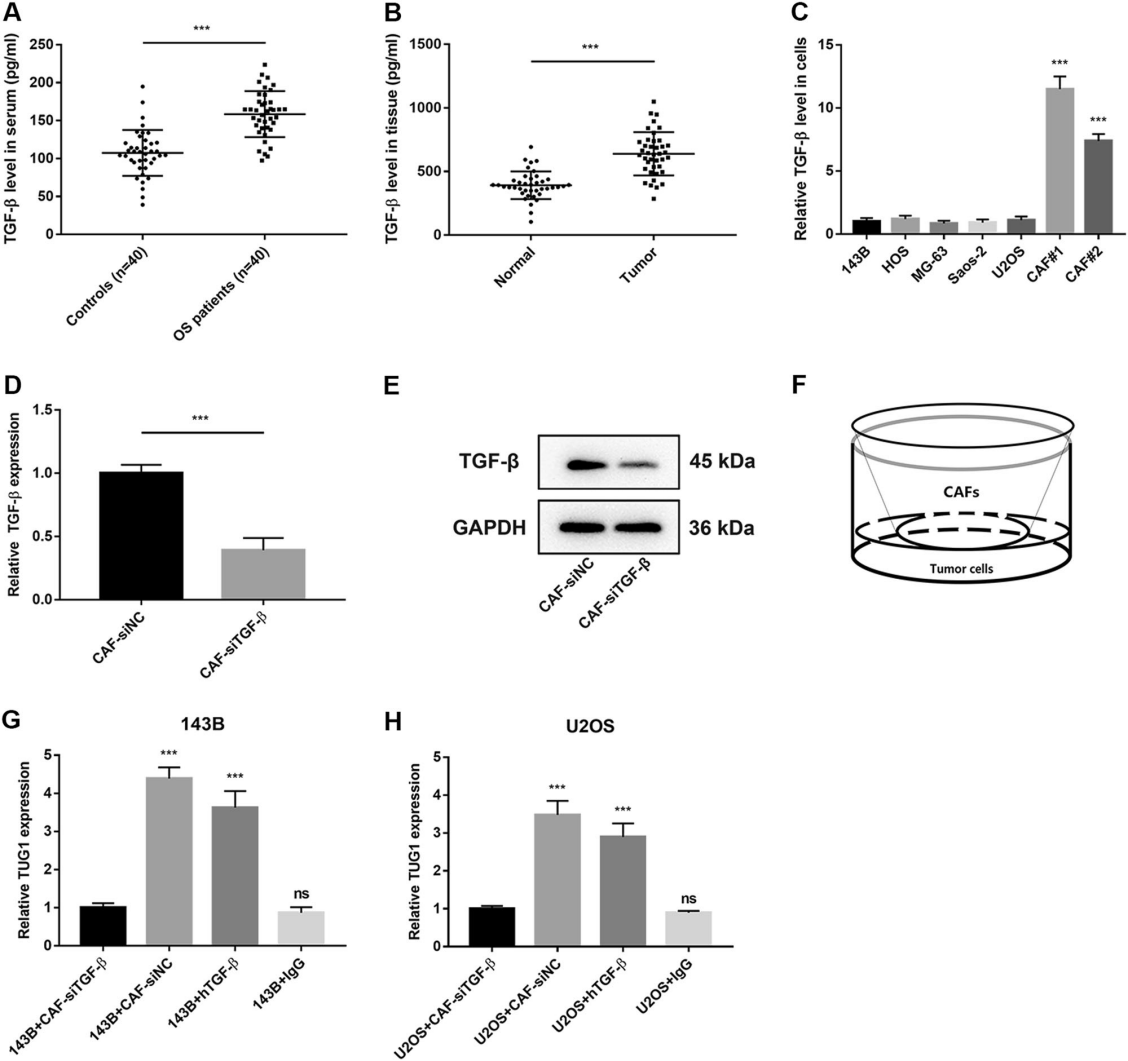
CAF-derived TGF-β promotes TUG1 expression in OS. **a** TGF-β expression levels in OS patients and the normal group were detected by ELISA. **b** TGF-β expression levels in OS and non-tumour tissues were detected by ELISA. **c** TGF-β expression levels in OS cell lines, OS CAFs, and normal fibroblasts were quantified by qRT-PCR. **d**, **e** Expression levels of TGF-β mRNA and protein in CAFs transfected with TGF-β siRNA (siTGF-β) were detected by qRT-PCR and western blot, respectively. **f** Diagram of the in vitro co-culture system. **g**, **h** Effect of CAF TGF-β knockdown and TGF-β on OS cell (143B and U2OS) TUG1 expression was determined using the in vitro co-culture system. ***P *<* 0.001; ns, not significant

Increasing evidence has demonstrated that cytokines from stroma cells, such as CAFs, could mediate the crosstalk between stroma cells and tumour cells, which is dysregulated for relevant gene expression in tumour cells^14^. TGF-β was also highly secreted from CAFs, and it was reported to activate lncRNA expression^15^. As TUG1 expression was to be found significantly higher in OS, we investigated the effect of TGF-β from OS-CAFs on TUG1 expression in OS. First, ELISA was performed to detect differences in serum TGF-β accumulation between OS patients and healthy controls. As shown in Fig. 2a (*P* < 0.05), TGF-β expression was significantly higher in OS patients’ serum than the healthy control group. Furthermore, TGF-β was detected in OS tissues and paired normal tissues, which these results were consistent with Fig. 2a (Fig. 2b, *P* *<* 0.05). Apart from OS tissues, we also investigated TGF-β expression in OS cell lines and CAFs, which showed that CAFs significantly produced more TGF-β than OS cells, and no differences were detected among OS cells (Fig. 2c, *P* *<* 0.05). Taken together, TGF-β was highly expressed in OS and mainly secreted from CAFs. TGF-β siRNAs (si-TGF-β) were used to silence the expression and secretion of TGF-β from CAFs, for which their effects on TGF-β mRNA and protein levels were validated (Fig. 2d, e, *P* *<* 0.05). As shown in Fig. 2f, an illustration demonstrated CAF transfected si-TGF-β co-culture with OS cells for 24 h. Following, TUG1 expression in OS cells was detected. With TGF-β expression modification confirmed, we detected TUG1 expression in both 143B-CAFs-siTGF-β and U2OS-CAFs-siTGF-β as much lower than in negative groups (Fig. 2g, h, *P* *<* 0.05). Additionally, 143B and U2OS cells treated with recombinant TGF-β protein expressed more TUG1 than 143B and U2OS cells alone (Fig. 2g, h, *P* *<* 0.05). Thus, CAFs-derived TGF-β promotes TUG1 expression in OS cells.

### TUG1 acts as miRNA sponge and negatively regulates miR-143-5p expression

**Fig. 3:**
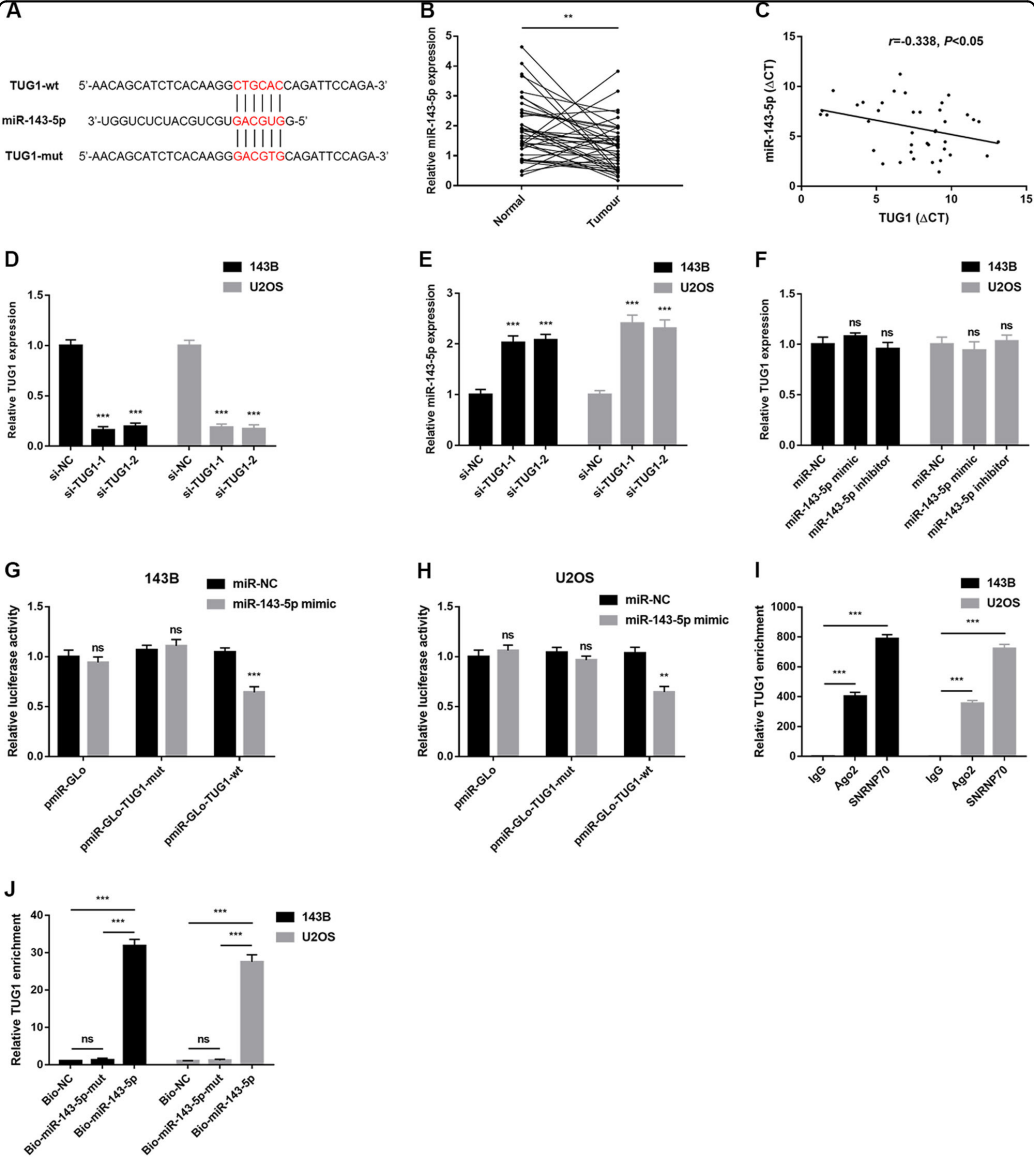
TUG1 acts as miRNA sponge and negatively regulates miR-143-5p expression. **a** Sequence alignment of miR-143-5p and TUG1. **b** Expression levels of miR-143-5p in OS and non-tumour tissues were detected by qRT-PCR. **c** Correlation between TUG1 and miR-143-5p expression was determined in OS tissues. **d** TUG1 expression levels in OS cells (143B and U2OS) transfected with TUG1 siRNA (si-TUG1) were detected by qRT-PCR. **e** Expression levels of miR-143-5p in OS cells (143B and U2OS) transfected with TUG1 siRNA (si-TUG1) were detected by qRT-PCR. **f** Expression levels of TUG1 in OS cells (143B and U2OS) transfected with miR-143-5p inhibitors were detected by qRT-PCR. **g**, **h** miR-143-5p reduced the activity of the luciferase reporter with the TUG1-specific sequence but not the mutant sequence. **i** Amount of TUG1 bound to Ago2 or IgG measured by RT-qPCR after RIP; IgG was used as a negative control and SNRNP70 was used as a positive control. **j** OS cells (143B and U2OS) were transfected with biotinylated wild-type miR-143-5p (Bio-miR-143-5p), biotinylated mutant miR-143-5p (Bio-miR-143-5p-mut), or biotinylated NC (Bio-NC). Forty-eight hours after transfection, cells were collected for the biotin-based pull-down assay. TUG1 expression levels were analysed by qRT-PCR. **P *<* 0.01; ***P *<* 0.001; ns not significant

Increasing evidence has demonstrated that lncRNAs could serve as miRNAs sponges to regulate target gene expression via competition with endogenous RNAs. Bioinformatics analysis (miRcode, http://www.mircode.org/index.php) was used to screen potential target binding miRNAs of TUG1 in OS. Among potential candidates, miR-143-5p was frequently reported as a tumour suppressor in OS^16–23^. The putative complementary sequences between TUG1 and miR-143-5p are illustrated in Fig. 3a. Subsequently, we performed a qRT-PCR assay to further assess the association between miR-143-5p and TUG1, which suggested that miR-143-5p was downregulated in OS tissues (Fig. 3b, *P* < 0.05). TUG1 was also negatively correlated with miR-143-5p in OS tissues (Fig. 3c, *P* *<* 0.05). Additionally, after assessing effects of si-TUG1 on silencing TUG1 expression (Fig. 3d, *P* *<* 0.05), we found that silencing of TUG1 via si-TUG1 transfection could lead to increased miR-143-5p accumulation compared with control groups (Fig. 3e, *P* *<* 0.05). However, upregulating or downregulating miR-143-5p had no influence on TUG1 expression (Fig. 3f, *P* > 0.05). To further explore whether the binding site was functional, we cloned the fragment, including the binding site as predicted by miRcode (Fig. 3a), into the pmiR-Glo vector as wild-type (pmiR-GLo-TUG1-wt) and mutated-type (pmiR-GLo-TUG1-wt). As shown in Fig. 3g, h, co-transfection of pmiR-GLo-TUG1-wt and miR-143-5p mimics greatly reduced luciferase activity compared with the pmiR-GLo-TUG1-wt + miR-NC group. Conversely, mutation of the miR-143-5p-binding site within TUG1 abrogated the inhibitory effect of miR-143-5p mimics on the reporter gene expression. Results from the RIP assay showed that TUG1 was preferentially enriched in Ago2-containing beads in OS cells (Fig. 3i), indicating that TUG1 is likely in the miR-143-5p RISC complex. Results from the miRNA pull-down demonstrated that miR-143-5p pulled down TUG1. However, miR-143-5p-mut with the mutated binding site of TUG1 failed to pull-down TUG1 (Fig. 3j), indicating that the recognition of miR-143-5p to TUG1 is in a sequence-specific manner. Together, our findings confirmed that miR-143-5p is regulated by TUG1 in OS cells through direct binding.

### TUG1 positively regulates HIF-1α via silencing of miR-143-5p

**Fig. 4:**
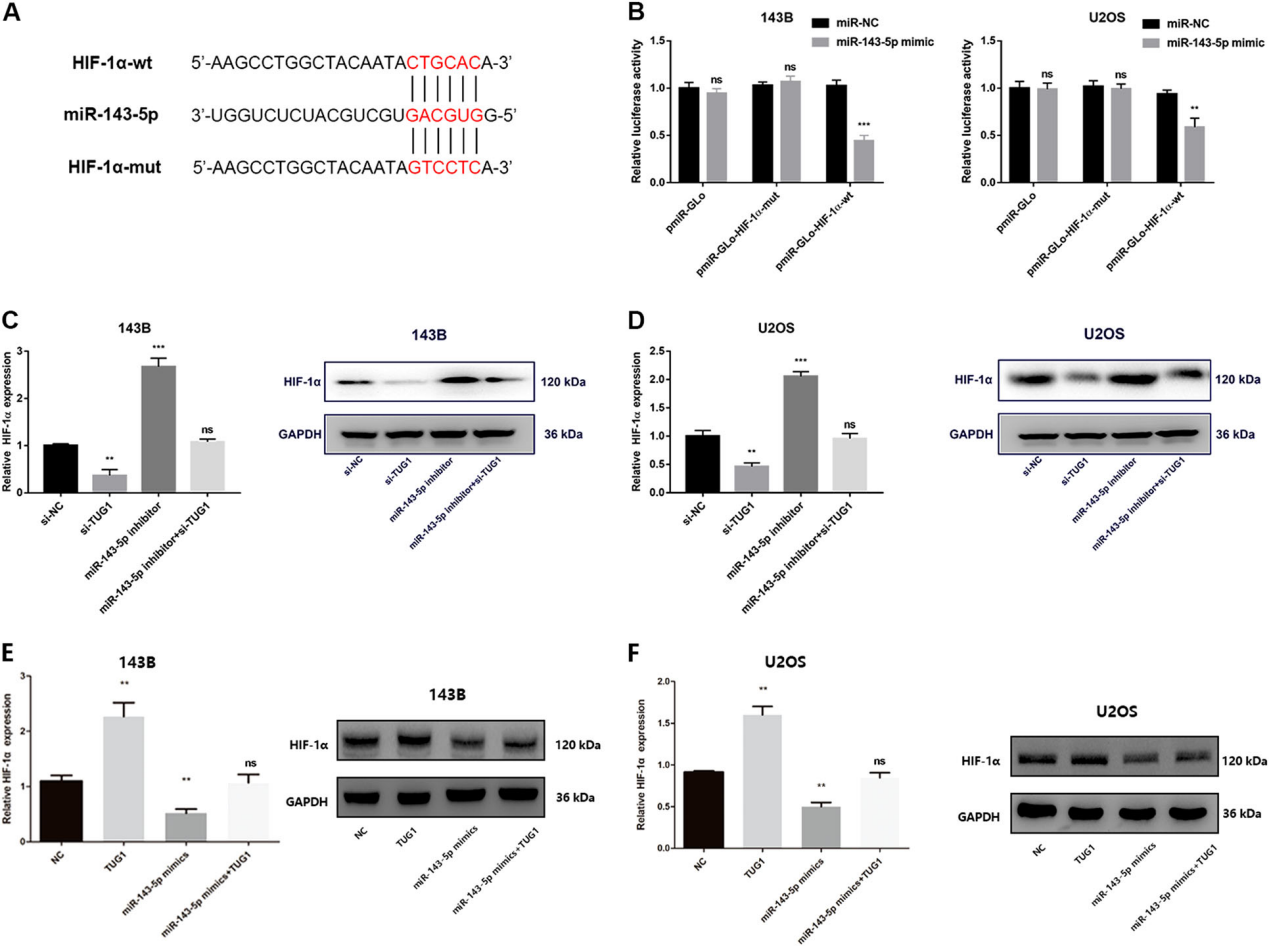
TUG1 positively regulates HIF-1α via miR-143-5p silencing. **a** Sequence alignment of miR-143-5p and HIF-1α was shown. **b** miR-143-5p reduced the activity of the luciferase reporter with the HIF-1α wild-type 3′UTR but not the mutant 3′UTR. **c**, **d** TUG1 knockdown decreased HIF-1α expression, which was reversed by the miR-143-5p inhibitor for mRNA and protein levels. **P *<* 0.01; ***P *<* 0.001; ns, not significant. **e**, **f** TUG1 upregulation increased HIF-1α expression, which was reversed by the miR-143-5p mimics for mRNA and protein levels. **P < 0.01; ***P < 0.001; ns, not significant

Bioinformatic analysis (TargetScan, http://www.targetscan.org; miRDB, http://www.mirdb.org) was performed to screen potential target genes of miR-143-5p. Among the overlapped potential candidates, HIF-1α was chosen as a target gene for its association with OS progression as reported^24–26^. As indicated in Fig. 4a, the putative binding sites between HIF-1α and miR-143-5p were predicted by TargetScan and miRDB. Dual luciferase reporter assay was performed to determine the negative effect of miR-143-5p on the HIF-1α 3′UTR. The results showed that co-transfection of pmiR-Glo-HIF-1-α wild-type and miR-143-5p mimics greatly reduced luciferase activity compared with the HIF-1α-wt + miR-143-5p NC group, whereas mutation of the miR-143-5p-binding site within HIF-1α abrogated the inhibitory effect of miR-143-5p mimics on the reporter gene expression (Fig. 4b, *P* *<* 0.05). Inhibition of miR-143-5p significantly increased HIF-1α mRNA and protein expression compared with negative control siRNAs; however, silencing of TUG1 inhibited HIF-1α expression. Additionally, transfection with si-TUG1 could significantly reverse the effect of miR-143-5p inhibitors on HIF-1α mRNA and protein expression (Fig. 4c, d, *P* *<* 0.05). To better establish the direct link between the TUG-1/miR-143-5p axis and HIF-1α, the effect of upregulated TUG-1 and miR-143-5p on HIF-1α expression was investigated. Upregulation of miR-143-5p could significantly impair HIF-1α mRNA and protein expression compared with the negative control. In contrast, increased TUG1 promoted HIF-1α expression, which was reversed by the miR-143-5p mimics at the mRNA or protein levels (Fig. 4e, f, *P* *<* 0.05). Taken together, TUG1 could serve as a sponge to competitively interact with miR-143-5p and reverse miR-143-5p-induced inhibition of HIF-1α expression.

### Inhibition of HIF-1α attenuates OS cell invasion and angiogenesis

**Fig. 5:**
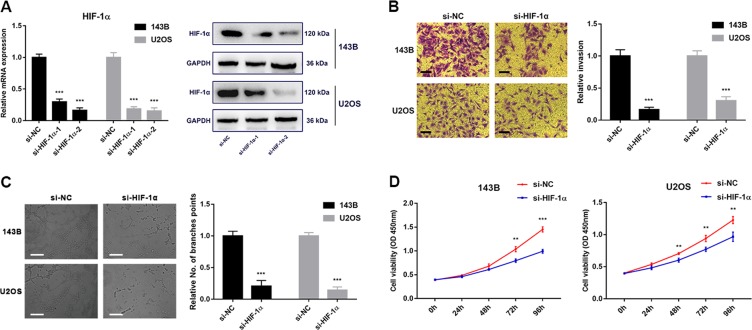
Inhibition of HIF-1α attenuates OS cell invasion and angiogenesis. **a** HIF-1α expression levels in OS cells (143B and U2OS) transfected with HIF-1α siRNAs (si HIF-1α) were detected by qRT-PCR and western blots. **b**, **c** HIF-1α knockdown inhibited OS cell migration (Scale bar = 50 μm), HUVECs angiogenesis (Scale bar = 100 μm), and proliferation. **d** HIF-1α knockdown inhibited OS cell proliferation. **P *<* 0.01; ***P *<* 0.001

With si-HIF-1α transfection, HIF-1α mRNA and protein expressions were significantly impaired, which was shown in Fig. 5a (*P* < 0.05). Further, we selected si-HIF-1α-2 in subsequent assays for its more effective inhibition. An invasion assay was performed to assess the effect of si-HIF-1α on OS cell invasion, and we found that inhibition of HIF-1α led to a significantly lower number of invasive cells than the negative control (Fig. 5b, *P* *<* 0.05). Furthermore, angiogenesis assay results showed that the tube networks formed by HUVECs were more extensive in the control group compared with the HIF-1α knockdown group (Fig. 5c, *P* *<* 0.05). Additionally, siRNAs targeting HIF-1α inhibited 143B or U2OS cell proliferation (Fig. 5d, *P* *<* 0.05). Taken together, HIF-1α, a target of miR-143-5p, positively regulates OS cell invasion and tube formation.

### TUG1 positively regulates OS cell invasion and angiogenesis via miR-143-5p silencing

**Fig. 6:**
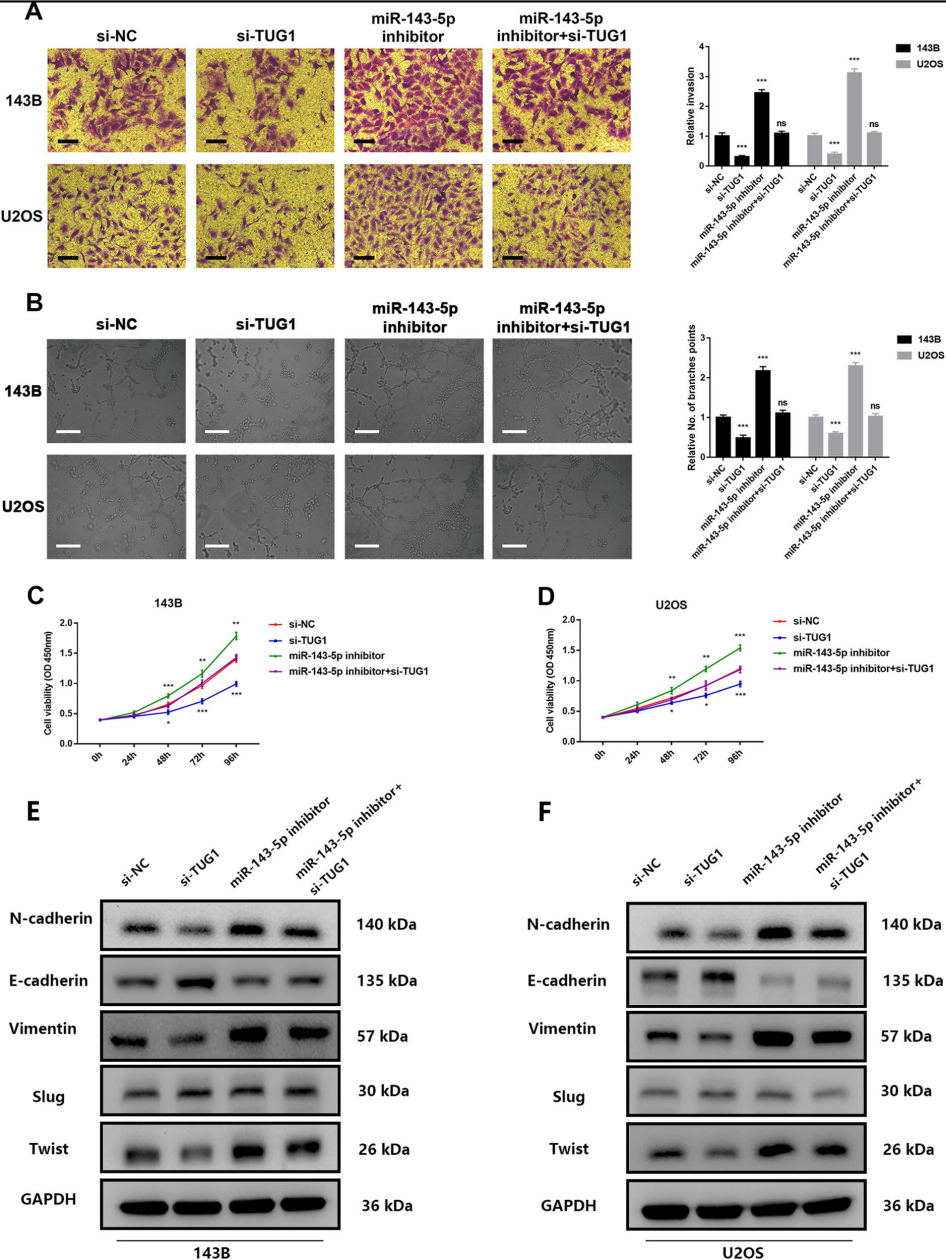
TUG1 positively regulates OS cell invasion and angiogenesis via miR-143-5p silencing. **a** TUG1 knockdown inhibited OS cell invasion, which was reversed by the miR-143-5p inhibitor. Scale bar = 50 μm. **b** TUG1 knockdown inhibited HUVECs angiogenesis, which was reversed by the miR-143-5p inhibitor. Scale bar = 100 μm. **c**, **d** TUG1 knockdown inhibited OS cell proliferation, which was reversed by the miR-143-5p inhibitor. **e**, **f** TUG1 knockdown upregulated the protein level of epithelial marker E-cadherin; it decreased the expression of mesenchymal markers N-cadherin and vimentin, and also decreased Twist, but not Slug, expression, which was reversed by the miR-144-3p inhibitor. *P < 0.05; **P < 0.01; ***P < 0.001; ns, not significant

Previously, the influence of HIF-1α on OS invasion and angiogenesis was determined. However, whether TUG1 could increase OS cell invasion ability via inhibiting miR-143-5p remained unclear. First, invasion cell numbers of siRNAs targeting the TUG1 group were significantly lower than the negative group in 143B and U2OS cells. In addition, compared to transfected miR-143-5p inhibitors group, the si-TUG1 co-transfected with miR-143-5p inhibitors group showed less invasion cells (Fig. 6a, *P* *<* 0.05). Consistently, an angiogenesis assay was performed, which exhibited similar results. Namely, miR-143-5p inhibitors could reverse the effect of si-TUG1 on OS cell invasion and angiogenesis (Fig. 6b, *P* *<* 0.05). Furthermore, the proliferation level in 143B and U2OS cells co-transfected with the miR-143-5p inhibitor and si-TUG1 was lower than in 143B and U2OS cells transfected with miR-143-5p inhibitors (Fig. 6c, d, *P* *<* 0.05). Additionally, EMT transition is a major process of tumour metastasis. Western blot analysis showed that silencing of TUG1 upregulated the protein level of epithelial marker E-cadherin, downregulated mesenchymal markers N-cadherin and vimentin expression, and could impair Twist but not Slug expression, which was reversed upon inhibition of miR-145-3p in both 143B and U2OS cells (Fig. 6e, f, *P* *<* 0.05).

### Silencing of TUG1 inhibits tumour growth, peritoneal spreading, and metastasis in vivo

**Fig. 7:**
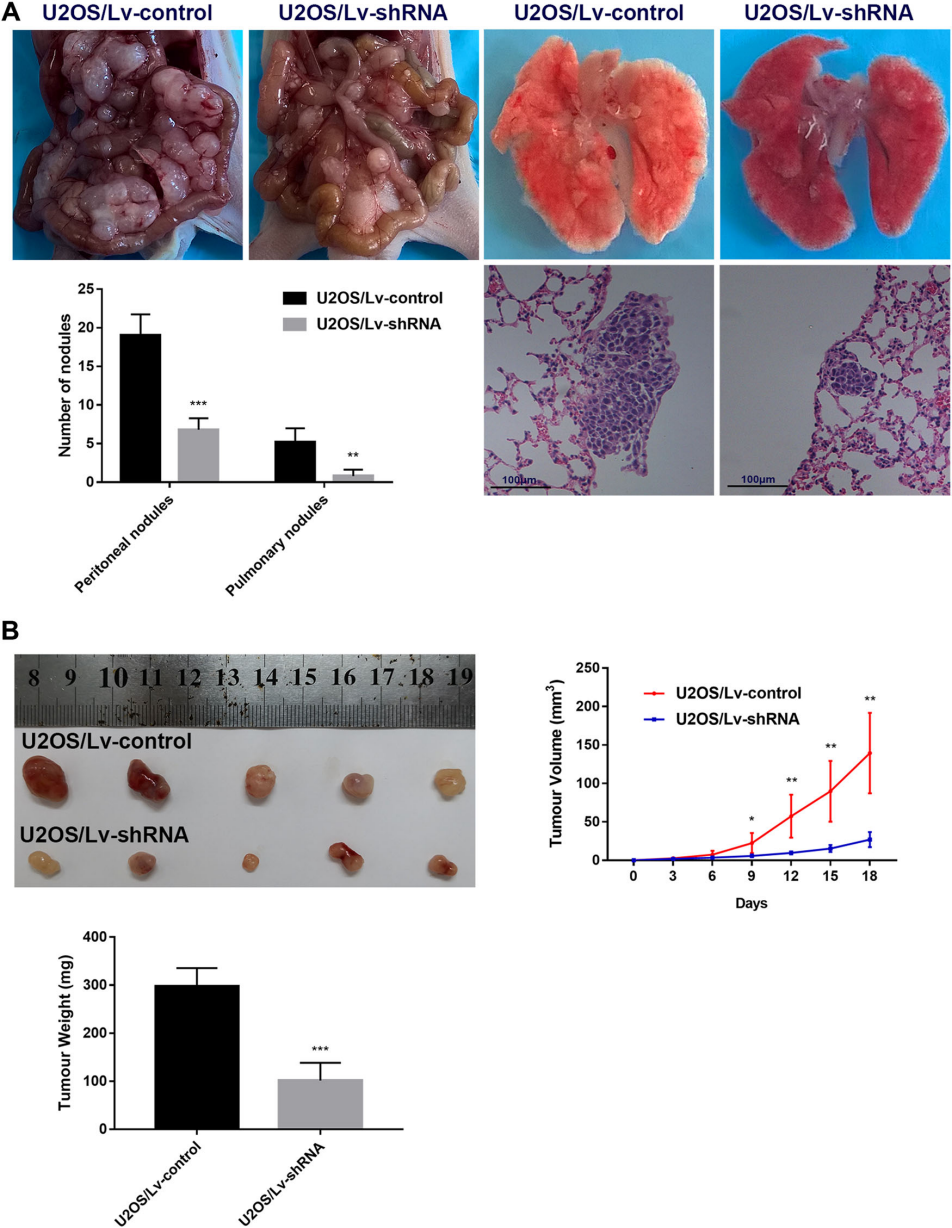
Silencing of TUG1 inhibits tumour growth, peritoneal spreading, and metastasis in vivo. **a** Representative photographs of peritoneal and pulmonary nodules in nude mice that resulted from peritoneally inoculated U2OS/Lv-control or U2OS/Lv-shRNA (TUG1). Scale bar = 100 μm. **b** Photographs of tumours in nude mice derived from U2OS/Lv-control or U2OS/Lv-shRNA (TUG1), and tumour volumes and weight were monitored. *P *<* 0.05; **P *<* 0.01; ***P *<* 0.001

We further tested whether silencing of TUG1 could repress tumour growth and metastasis in vivo. Injection of U2OS cells was performed in nude mice. There were significantly less visible peritoneal and pulmonary nodules in U2OS/Lv-shRNA compared to the control group (Fig. 7a, *P* *<* 0.05). Similarly, the U2OS cells with Lv-shRNA group generated tumours of smaller volume and weight (Fig. 7b, *P* *<* 0.05) than those generated by U2OS cells with Lv-NC. We concluded that TUG1 knockdown promoted tumourigenesis, peritoneal spread, and metastasis of OS in vivo.

## Discussion

Recently, a growing number of studies have reported the functional roles of lncRNAs in tumourigenesis and progression of OS^27–31^. TUG1 is located on human chromosome 22q12.2 and was initially detected for its upregulation in mouse retinal cells under taurine treatment^32^. TUG1 was originally reported for its functional role in the differentiation of rodent retina, followed by recent reports of its cancer-promoting effect in diverse carcinomas. Cai et al. suggested that TUG1 promoted tumour-induced angiogenesis and upregulated VEGF expression by downregulating miR-299 in human glioblastoma^33^. Dong et al. proposed that the TUG1/miR-34a-5p/VEGFA pathway potentiated hypervascularity and hepatoblastoma progression^34^. Zhang et al. reported that TUG1 regulated gastric cancer cell growth by epigenetically repressing p57 expression^35^. Ma et al. found that TUG1 promoted gallbladder carcinoma cell proliferation and metastasis by downregulating miR-300^36^. Additionally, TUG1 has been reported to act as an oncogene in OS^37–39^.

The present study, based on previous studies, especially emphasised the potential effects of TUG1 in OS angiogenesis and metastasis. As a tumour promoter in OS progression, TUG1 was significantly overexpressed in tumour tissues and cell lines. Clinically, elevated TUG1 levels in OS patients significantly correlated with tumour size, distant metastasis, TNM stage, and overall and recurrence-free survival. To this end, we performed transwell invasion, tube formation, and CCK-8 assays at the cellular level to validate that TUG1 knockdown significantly inhibited OS cell angiogenesis, proliferation, and invasion. Correspondingly, the results of the in vivo metastasis and tumourigenesis assays reinforced this above-mentioned conclusion.

Further, we attempted to detail the mechanism by which TUG1 expression levels are increased in OS. CAFs, a major stromal cell type of cancer, play a vital role in facilitating malignant progression and could be a novel therapeutic tool^40^. Through secretion of various cytokines (such as TGF-β, IL-6, CXCL14, etc.) into the tumour microenvironment, CAFs endow cancer cells with proliferative, invasive, and angiogenic properties^41,42^. As an abundant cytokine and well-established EMT inducer in the tumour microenvironment, TGF-β has been reported to play vital roles in OS progression^43^. Our study showed that the relative expression level of TGF-β in CAFs was significantly higher than that in OS cells, which, at least, partly proved this above. Moreover, we found that compared to their controls, TGF-β levels in the serum and tissues of OS patients were significantly increased. Furthermore, we found that TGF-β secreted by CAFs was associated with TUG1 upregulation, which indicated that the function of TGF-β in promoting OS malignant progression might be partly through induction of TUG1 overexpression.

To elucidate the mechanism by which TUG1 works as an oncogene in OS, we performed dual-luciferase reporter, RIP, and biotin-labelled miRNA pull-down assays and demonstrated that TUG1 could competitively bind to miR-143-5p and regulate its target gene HIF-1α expression. Although the ‘sponge’ role of TUG1 for miRNAs had been reported several times, we first detected and confirmed the interaction between miR-143-5p and TUG1. Some previous studies suggested that miR-143-3p was significantly downregulated and functioned as a tumour suppressor in some cancers^21,44–46^. However, the effects of miR-143-5p on the biological characteristics of cancer cells were rarely explored. He et al. suggested that miR-143-5p was significantly downregulated in gallbladder cancer tissues and inhibited cell metastasis by targeting HIF-1α^47^. Our finding demonstrated that miR-143-5p was also significantly downregulated and negatively correlated with the corresponding expression level of TUG1 in OS tissues. While, as expected, TUG1 knockdown significantly upregulated the miR-143-5p expression level, which might be associated with Drosha and Dicer^48^. Given these findings, we propose that TUG1 could be a ceRNA for miR-143-5p in OS.

HIF-1α, a transcription factor, is often induced by an intratumoural hypoxic microenvironment and is closely related to tumour angiogenesis, metastasis, and growth^49^. The formation of new blood vessels is related to increases in angiogenic molecules and decreases in antiangiogenic molecules^50^. Under hypoxic conditions, HIF-1α can stimulate overexpression of the angiogenic element VEGF, which induces the differentiation, proliferation, and chemotaxis of endothelial cells^51^. Cai et al. also suggested that HIF-1α knockdown inhibited cell migration, invasion, and EMT progression by directly targeting Slug in gallbladder cancer under hypoxic conditions^11^. In this study, we confirmed the functional role of HIF-1α in OS cell proliferation, metastasis, and angiogenesis.

**Fig. 8:**
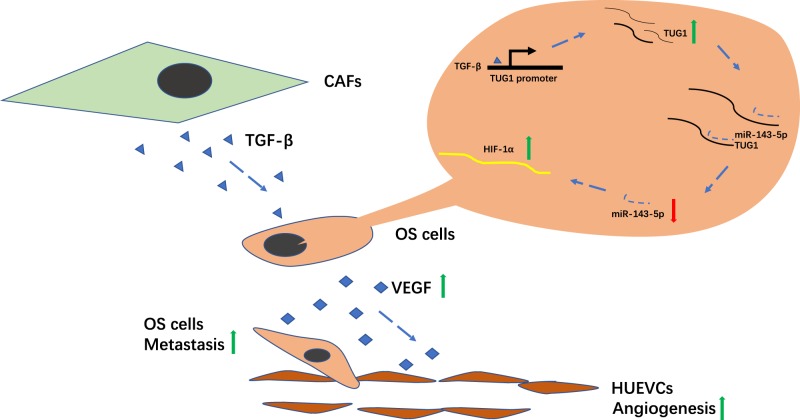
Proposed model of TUG1 function in OS cells. Crosstalk between OS cells and their stromal CAFs contributed to OS progression through the TUG1/miR-143-5p/HIF-1α pathway

In summary, we identified the crosstalk between OS cells and their stromal CAFs and its contribution to OS progression through the TUG1/miR-143-5p/HIF-1α pathway (Fig. 8). TUG1 acted as a ceRNA to abrogate the endogenous effect of miR-143-5p, which suppressed HIF-1α expression. Our study not only emphasised the effects of TUG1 in OS, but also demonstrated a novel axis by which TUG1 regulated OS cell metastasis, angiogenesis, and proliferation. Therefore, TUG1 might be a prognostic indicator and therapeutic target for OS.

## Supplementary information

Supplementary Figure 1

Supplementary Table 1

Supplementary figure legends

## References

[1] Miller BJ, Gao Y, Duchman KR (2017). Socioeconomic measures influence survival in osteosarcoma: an analysis of the National Cancer Data Base. Cancer Epidemiol..

[2] Mirabello L, Troisi RJ, Savage SA (2009). International osteosarcoma incidence patterns in children and adolescents, middle ages and elderly persons. Int. J. Cancer.

[3] Luetke A, Meyers PA, Lewis I, Juergens H (2014). Osteosarcoma treatment - where do we stand? A state of the art review. Cancer Treat. Rev..

[4] Paduch R (2016). The role of lymphangiogenesis and angiogenesis in tumor metastasis. Cell. Oncol..

[5] Maishi N, Hida K (2017). Tumor endothelial cells accelerate tumor metastasis. Cancer Sci..

[6] Yeung KT, Yang J (2017). Epithelial-mesenchymal transition in tumor metastasis. Mol. Oncol..

[7] Zheng H, Li W, Kang Y (2016). Tumor-Stroma Interactions in Bone Metastasis: Molecular Mechanisms and Therapeutic Implications. Cold Spring Harb. Symp. Quant. Biol..

[8] Su W, Xie W, Shang Q, Su B (2015). The Long noncoding RNA MEG3 is downregulated and inversely associated with VEGF levels in osteoarthritis. BioMed. Res. Int..

[9] Zhu Y (2016). HULC long noncoding RNA silencing suppresses angiogenesis by regulating ESM-1 via the PI3K/Akt/mTOR signaling pathway in human gliomas. Oncotarget.

[10] Li N, Shi K, Kang X, Li W (2017). Prognostic value of long non-coding RNA TUG1 in various tumors. Oncotarget.

[11] Cai, Q. *et**al*. Long non-coding RNA LINC00152 promotes gallbladder cancer metastasis and epithelial-mesenchymal transition by regulating HIF-1alpha via miR-138. *Open Biol.* **7**. 10.1098/rsob.160247 (2017). 10.1098/rsob.160247 PMC5303272 28077595

[12] Wang Y, Zhang R, Cheng G, Xu R, Han X (2018). Long non-coding RNA HOXA-AS2 promotes migration and invasion by acting as a ceRNA of miR-520c-3p in osteosarcoma cells. Cell Cycle.

[13] Tsai HC (2017). WISP-1 positively regulates angiogenesis by controlling VEGF-A expression in human osteosarcoma. Cell death & Dis..

[14] Avgustinova A (2016). Tumour cell-derived Wnt7a recruits and activates fibroblasts to promote tumour aggressiveness. Nat. Commun..

[15] Yuan JH (2014). A long noncoding RNA activated by TGF-beta promotes the invasion-metastasis cascade in hepatocellular carcinoma. Cancer Cell.

[16] Dong X (2017). MiR-143 regulates the proliferation and migration of osteosarcoma cells through targeting MAPK7. Arch. Biochem. Biophys..

[17] Hirahata M (2016). PAI-1, a target gene of miR-143, regulates invasion and metastasis by upregulating MMP-13 expression of human osteosarcoma. Cancer Med..

[18] Li WH (2016). MicroRNA-143 promotes apoptosis of osteosarcoma cells by caspase-3 activation via targeting Bcl-2. Biomed. Pharmacother..

[19] Liu H, Wang H, Liu H, Chen Y (2015). Effect of miR-143 on the apoptosis of osteosarcoma cells. Int. J. Clin. Exp. Pathol..

[20] Osaki M (2011). MicroRNA-143 regulates human osteosarcoma metastasis by regulating matrix metalloprotease-13 expression. Mol. Ther..

[21] Sun X (2018). miR-143-3p inhibits the proliferation, migration and invasion in osteosarcoma by targeting FOSL2. Sci. Rep..

[22] Wang Q, Cai J, Wang J, Xiong C, Zhao J (2014). MiR-143 inhibits EGFR-signaling-dependent osteosarcoma invasion. Tumour Biol..

[23] Zhang H (2010). microRNA-143, down-regulated in osteosarcoma, promotes apoptosis and suppresses tumorigenicity by targeting Bcl-2. Oncol. Rep..

[24] Guan G (2015). The HIF-1alpha/CXCR4 pathway supports hypoxia-induced metastasis of human osteosarcoma cells. Cancer Lett..

[25] Lv F (2016). HIF-1alpha silencing inhibits the growth of osteosarcoma cells by inducing apoptosis. Ann. Clin. Lab. Sci..

[26] Wang X, Liang X, Liang H, Wang B (2018). SENP1/HIF-1alpha feedback loop modulates hypoxia-induced cell proliferation, invasion, and EMT in human osteosarcoma cells. J. Cell. Biochem..

[27] Wang Y (2018). Long noncoding RNA DANCR, working as a competitive endogenous RNA, promotes ROCK1-mediated proliferation and metastasis via decoying of miR-335-5p and miR-1972 in osteosarcoma. Mol. Cancer.

[28] Wang Y (2016). Long non-coding RNA LINC00161 sensitises osteosarcoma cells to cisplatin-induced apoptosis by regulating the miR-645-IFIT2 axis. Cancer Lett..

[29] Ye K (2017). Long Noncoding RNA GAS5 suppresses cell growth and epithelial-mesenchymal transition in osteosarcoma by regulating the miR-221/ARHI Pathway. J. Cell. Biochem..

[30] Zhang CL, Zhu KP, Ma XL (2017). Antisense lncRNA FOXC2-AS1 promotes doxorubicin resistance in osteosarcoma by increasing the expression of FOXC2. Cancer Lett..

[31] Zhu KP, Ma XL, Zhang CL (2017). LncRNA ODRUL contributes to osteosarcoma progression through the miR-3182/MMP2 Axis. Mol. Ther.: J. Am. Soc. Gene Ther..

[32] Young TL, Matsuda T, Cepko CL (2005). The noncoding RNA taurine upregulated gene 1 is required for differentiation of the murine retina. Curr. Biol.: CB.

[33] Cai, H. et al. Long non-coding RNA taurine upregulated 1 enhances tumor-induced angiogenesis through inhibiting microRNA-299 in human glioblastoma. *Oncogene*10.1038/onc.2016.212 (2016). 10.1038/onc.2016.212 27345398

[34] Dong R (2016). Targeting long non-coding RNA-TUG1 inhibits tumor growth and angiogenesis in hepatoblastoma. Cell Death & Dis..

[35] Zhang E (2016). Increased expression of long noncoding RNA TUG1 predicts a poor prognosis of gastric cancer and regulates cell proliferation by epigenetically silencing of p57. Cell Death & Dis..

[36] Ma F (2017). Long non-coding RNA TUG1 promotes cell proliferation and metastasis by negatively regulating miR-300 in gallbladder carcinoma. Biomed. & Pharmacother..

[37] Cao J, Han X, Qi X, Jin X, Li X (2017). TUG1 promotes osteosarcoma tumorigenesis by upregulating EZH2 expression via miR-144-3p. Int. J. Oncol..

[38] Li, Y., Zhang, T., Zhang, Y., Zhao, X. & Wang, W. Targeting the FOXM1-regulated long non-coding RNA TUG1 in osteosarcoma. *Cancer Sci.*10.1111/cas.13765 (2018). 10.1111/cas.13765 PMC6172046 30099814

[39] Wang Y (2017). Long non-coding RNA TUG1 promotes migration and invasion by acting as a ceRNA of miR-335-5p in osteosarcoma cells. Cancer Sci..

[40] De Vlieghere E, Verset L, Demetter P, Bracke M, De Wever O (2015). Cancer-associated fibroblasts as target and tool in cancer therapeutics and diagnostics. Virchows Arch.: Int. J. Pathol..

[41] Augsten M (2009). CXCL14 is an autocrine growth factor for fibroblasts and acts as a multi-modal stimulator of prostate tumor growth. Proc. Natl Acad. Sci. USA.

[42] Wu X (2017). IL-6 secreted by cancer-associated fibroblasts promotes epithelial-mesenchymal transition and metastasis of gastric cancer via JAK2/STAT3 signaling pathway. Oncotarget.

[43] Lv Z (2014). Bone morphogenetic protein 9 regulates tumor growth of osteosarcoma cells through the Wnt/beta-catenin pathway. Oncol. Rep..

[44] Jin YP (2018). miR-143-3p targeting of ITGA6 suppresses tumour growth and angiogenesis by downregulating PLGF expression via the PI3K/AKT pathway in gallbladder carcinoma. Cell Death Dis..

[45] Li D (2017). miR-143-3p targeting LIM domain kinase 1 suppresses the progression of triple-negative breast cancer cells. Am. J. Transl. Res..

[46] Shi H (2018). MiR-143-3p suppresses the progression of ovarian cancer. Am. J. Transl. Res..

[47] He M (2017). MiR-143-5p deficiency triggers emt and metastasis by targeting HIF-1alpha in gallbladder cancer. Cell. Physiol. Biochem..

[48] Wang K (2014). MDRL lncRNA regulates the processing of miR-484 primary transcript by targeting miR-361. PLoS Genet..

[49] Nepal M (2012). Anti-angiogenic and anti-tumor activity of Bavachinin by targeting hypoxia-inducible factor-1alpha. Eur. J. Pharmacol..

[50] De Palma M, Biziato D, Petrova TV (2017). Microenvironmental regulation of tumour angiogenesis. Nat. Rev. Cancer.

[51] Ferrara N (2002). VEGF and the quest for tumour angiogenesis factors. Nat. Rev. Cancer.

