# Supplementary figures and images for: Long non-coding RNA Taurine upregulated gene 1 promotes osteosarcoma cell metastasis by mediating HIF-1α via miR-143-5p
Source: Cell Death Dis. 2019 Mar 25;10(4):280. doi: 10.1038/s41419-019-1509-1 (PMC6433912; doi:10.1038/s41419-019-1509-1)

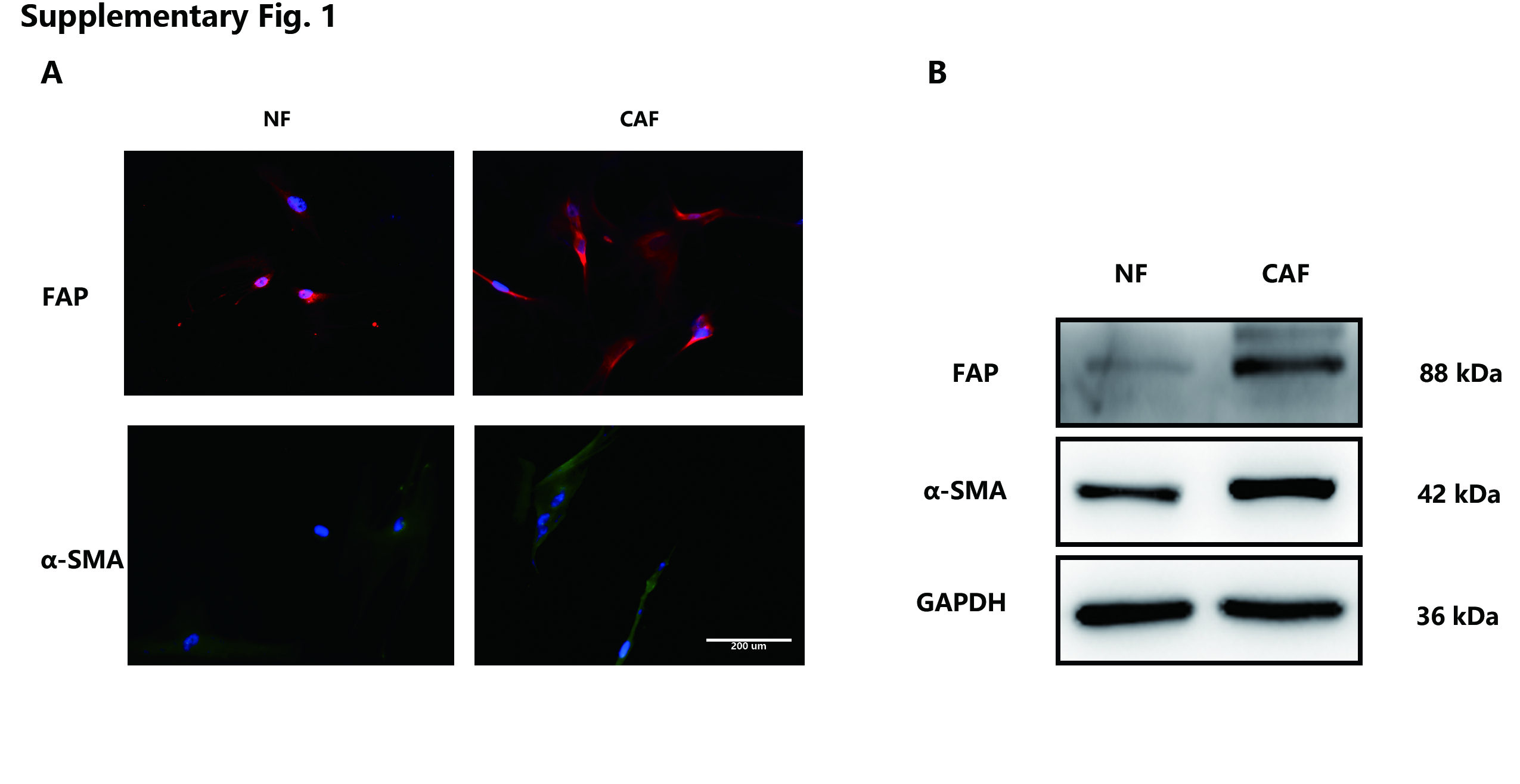

Supplement: Supplementary file 1 — Supplementary Figure 1 [file 41419_2019_1509_MOESM1_ESM.tif]
