# Supplementary material for: Long non-coding RNA Taurine upregulated gene 1 promotes osteosarcoma cell metastasis by mediating HIF-1α via miR-143-5p
Source: Cell Death Dis. 2019 Mar 25;10(4):280. doi: 10.1038/s41419-019-1509-1 (PMC6433912; doi:10.1038/s41419-019-1509-1)
Supplement: Supplementary file 3 — Supplementary figure legends [file 41419_2019_1509_MOESM3_ESM.docx]

**Supplementary Figure 1.** Verification of CAFs and NFs derived from OS patients. (A) Primary fibroblastic population isolated from human OS tissue and adjacent non-cancerous tissue from the same patients was immunostained with anti-a-SMA and anti-FAP antibodies. Scale bar, 200 μm. (B) a-SMA and FAP mRNA levels in both CAFs and NFs were quantified by qRT-PCR.
